# Feed Intake Parameters of Horses Fed Soaked or Steamed Hay and Hygienic Quality of Hay Stored following Treatment

**DOI:** 10.3390/ani11092729

**Published:** 2021-09-18

**Authors:** Maren Glatter, Mandy Bochnia, Monika Wensch-Dorendorf, Jörg Michael Greef, Annette Zeyner

**Affiliations:** 1 Group Animal Nutrition, Martin Luther University Halle-Wittenberg, 06120 Halle (Saale), Germany; maren.glatter@landw.uni-halle.de (M.G.); mandy.bochnia@landw.uni-halle.de (M.B.); 2 Biometrics and Informatics in Agriculture Group, Martin Luther University Halle-Wittenberg, 06120 Halle (Saale), Germany; monika.dorendorf@landw.uni-halle.de; 3 Federal Research Center for Cultivated Plants, Crop and Soil Science, Julius Kuehn Institute, 38104 Braunschweig, Germany; joerg-michael.greef@julius-kuehn.de

**Keywords:** horses, hay, microbial content, hygienic quality, chewing, feed intake

## Abstract

**Simple Summary:**

Dusty hay is particularly harmful to horses with equine asthma, where the dust mainly consists of microbial deposits in addition to abiotic ones. Soaking and steaming hay can improve its hygienic quality by rinsing off dust, but also reducing the viability of microorganisms. In this study, we investigated whether the treated hay remains stable during subsequent storage, and how the horses’ feed intake as well as chewing activity change with treated hay. Microbial counts were determined by culture methods in meadow hay before and after soaking or steaming, and subsequent storage at 10 and 25 °C for 6, 12 and 24 h. Chewing activity was monitored while horses consumed native or treated hay. Steaming effectively reduced yeasts and typical mold. Steamed hay was almost stable during storage, but storing soaked hay increased yeasts, and typical bacteria and mold. The intake of soaked hay was characterized by a particularly low consumption rate and high chewing intensity, but these per se positive effects seemed to be biased by a lower acceptance. However, steaming can be used to reduce counts of viable microorganisms. The feeding of soaked hay is recommended directly after treatment, to avoid hygienic problems.

**Abstract:**

Horses suffering from equine asthma must consume low-dust forage, with soaking and steaming being suitable methods of hay treatment. The impacts of this treated hay’s subsequent storage and effects on the horses’ chewing activity are largely unknown. Meadow hay was soaked (10–15 °C, 15 min) or steamed (100 °C, 60 min). Microbial counts (colony forming units (CFU)) were determined by culture before and after soaking or steaming, and subsequent storage at 10 and 25 °C for 6, 12 and 24 h (three replicates each). Six horses were fed native, soaked and steamed hay, according to a cross-over design, and chewing parameters were measured. Steaming reduced (*p* < 0.05) typical mold vs. soaking (0 vs. 50 CFU/g) and yeasts vs. native and steamed hay (0 vs. 102 and 90 CFU/g). Storing soaked hay elevated bacteria, mold, and yeasts (*p* < 0.05). Within the first 60 min of hay intake, the steamed hay and soaked hay were eaten slower (19.5 and 21.5 g dry matter/min, respectively; *p* < 0.05) and the steamed hay was chewed more intensely (steamed hay: 3537; native: 2622; and soaked: 2521 chewing cycles/kg dry matter, *p* < 0.05). Steaming particularly improves the hygienic quality of hay. Soaked hay is not stable when stored and is less accepted by horses.

## 1. Introduction

In horse nutrition, the hygienic status of feedstuffs is essential to maintaining the health and performance of the animals. In addition to the more or less manageable hygienic problems, the feeding of “long-stemmed forage”, such as hay, in general, can help to satisfy the animal´s innate need to chew [1]. Previous studies revealed that the chewing parameters in horses can vary between feedstuffs (concentrate or roughage; [2]), but also between concentrates (oat grains and compound feed, such as muesli or pelleted; [3]) and forage (alfalfa, timothy, and fresh grass; [4]), as well as between special treatments of concentrates (e.g., native, crushed and rolled cereal grains and compound feeds with different pellet diameters; [3,5,6]), with various effects on tooth wear, saliva production, and stomach health. The effect of roughage on feed intake (FI) behavior if, e.g., steamed hay is offered to horses, was also evaluated in recent studies. For example, studies on voluntary FI have reported that the intake of steamed hay was higher than that of haylage and native or soaked hay [7,8]. Earing et al. (2013) observed a significant increase in dry matter (DM) intake following steaming hay, with little mold [9]. In addition to the reduction in molds and other microbes, the higher moisture after steaming may increase the DM intake. Considering the level of DM in roughages, horses seem to prefer discreet higher moisture contents if they have the choice. A choice study conducted by Mueller and Udén (2007) observed the highest DM intakes for horses offered silage compared to haylage or hay [10]. However, not only the DM content, but also the conservation method (smell and taste), the nutrient content, and other characteristics of the forage affected the preference and chewing parameters. Using three forages of similar DM content, Janis et al. (2010) revealed significant differences between alfalfa, timothy, and a mixed hay diet, regarding the chewing frequency (CFR; number of chews per time) and the consumption rate (CR; amount of ingested food per time) when expressed on a DM basis, but not when expressed on a fiber basis [4]. Therefore, the authors concluded that with higher fiber contents, the CR and chewing frequency decreased, indicating that the horses chew the feed more thoroughly. To what extent soaking or different treating methods and their products affect the acceptance, and thereby specific parameters of FI (e.g., g DM/kg body weight (bw)) and chewing (CR, CFR, and chewing intensity (CI; in chewing cycles (CC)/kg DM)) in horses, was not examined in the cited studies. Hay can maintain normal feeding behavior, and promote dental and intestinal health [1]. An additional positive effect of soaking, which prolonged ingestion time and increased chewing intensity, as observed in the present study, can be viewed as advantageous. So, the percentage of feed intake time can be increased without additional feed, which is beneficial for horses with EMS or other metabolic disorders [11]. Additionally, the amount of saliva might be higher during longer chewing times, which has a positive effect on gastrointestinal health [6]. Further, Argo et al. (2015) demonstrated the use of soaked grass hay to promote weight loss in the management of equine metabolic syndrome (EMS) and obesity. The authors corrected the DM provision of soaked hay compared to native hay after evaluating the nutrient composition. [11]. The reduced acceptance of the treated hay may have influenced ingestion behavior and led to restrained intake.

Hay is part of the most basic rations and due to the nature of the production process and the actual environmental conditions (especially the weather), it is prone to being contaminated with bacteria, molds, fungal spores, organic dust, plant particles (e.g., small parts of flowers, stems, and broken leaves), and insect fragments, which can have an allergenic impact on horses [12]. The uptake of small particles (≤5 µm, alveolar) can cause hypersensitivity in the horses lungs, which might lead to respiratory diseases, such as equine asthma (formerly, chronic obstructive pulmonary disease (COPD); [9,13,14]). Moreover, the oral ingestion of bacteria and endotoxins, and/or molds and mycotoxins as their metabolites, is assumed to disturb the gastrointestinal fermentation processes and lead to colic [15,16,17]. In the literature, different treatments are described to improve the hygienic quality of hay. Here, soaking and/or steaming seem to be the most effective methods to reduce alveolar particles and microbial pollution [17,18,19]. The soaking and steaming of hay both resulted in a reduction in airborne respirable particles by ~100% [16]. Furthermore, mold spores were reduced by 73% after soaking, whereas steaming led to a reduction of ~100% [14,16,20]. The steaming of hay is reported to reduce the contamination with bacteria by 98–99% [14,17]. Conversely, soaking hay increased the bacterial counts up to 1.5 times compared to native hay [16,20]. As such, the water remaining following hay soaking had a higher polluting potential (concerning the waste water) than that following hay steaming [18]. The potential risk factor for environmental pollution seems to be higher with the soaking treatment, in contrast to steaming.

The washing out of water-soluble carbohydrates (WSCs) and minerals by soaking and the less-intense steaming of hay is applied in the management of metabolic disorders in horses, such as equine metabolic syndrome, polysaccharide storage myopathy (PSSM), hyperkalemic periodic paralysis, and pituitary pars intermedia dysfunction [11,21,22]. Soaking (for 15 min, 30 min, 1 h, 7 h, 12 h, or 16 h) reduced the contents of WSCs and ash, depending on the soaking duration, by 25–47% and 10–58%, respectively [11,23]. Martinson et al. (2012) recommended a limited soaking duration of 15–30 min for horses suffering from laminitis, PSSM, and equine asthma, because longer timeframes resulted in nutrient deficiencies, which must be compensated [22]. The steaming of hay has been widely reported to maintain minerals, trace elements, and crude protein, and only slightly reduce the content of WSCs compared to soaking [17,19]. 

The aim of the current study was to investigate how the soaking and steaming of hay influences the FI and chewing parameters in horses. Furthermore, the objective of this study was to test the impact of the treatments (soaking or steaming) and subsequent storage of hay on the microbial quality. We hypothesized that (1) altered water content and texture/grip for the treated hay would affect the FI and chewing parameters, and (2) the storage of soaked hay would increase microbial counts to a higher degree than that of native or even steamed hay.

## 2. Materials and Methods

The study was conducted using hay from middle Germany (meadow hay, dominated by Gramineae, first cut, end of blossom) from one batch both for the measurement of the FI, several feed intake parameters and the laboratory analysis (nutrient composition and hygienic quality). For sample management see [23].

**Figure 1:**
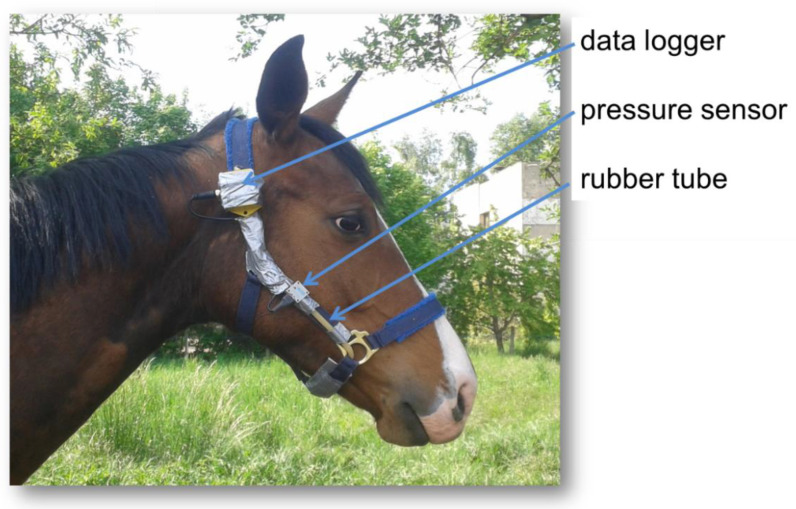
Modified halter equipped with measuring instruments for the detection of chewing parameters.

For the detection of the FI and chewing parameters, 6 adult, clinical healthy and dental fit warmblood mares (age 11 ± 2.7 years, bw 536 ± 36.0 kg, body condition score (BCS) 5.3 ± 0.33/9 according to Kienzle and Schramme (2004) [24]) were stabled in boxes (straw bedding) with individual paddocks (free access to dry-lot paddock) and fed hay (ranging from 4.2 to 4.8 kg original matter (OM) hay per meal according to metabolizable energy (ME) = 0.52 MJ ME kg bwt^0.75^ * d^−1^ (GfE, 2014 [25]), based on the calculated energy content from the feed analysis of the native hay. The hay was fed in equal meals at 8:00 a.m. and 3:00 p.m. The horses had free access to thermally controlled water bowl (~10 °C water temperature) and a salt stick containing sodium chloride only. Steaming was conducted using a common hay steamer (Haygain HG 2000, Farm & Stable, West Sussex, UK) as specified by the manufacturer. For treatment, but not for feeding, the hay was individually weighted into hay nets (mesh size = 5 cm) and steamed immediately before feeding. The steaming process lasted 1 h with a target temperature of 100 °C inside the hay. For soaking, the hay was also individually weighed into hay nets (not for feeding) and placed in a metal tube (approximately 488 L holding capacity) in the stable (ambient temperature 5–10 °C). Water was added directly from the water pipe (12–15 °C) into the metal tube and the hay was soaked for 15 min, drained for 10 min and fed immediately afterwards. During the adaptation period, the horses were additionally allowed to free range for 30 min with 2–3 conspecifics. According to a cross-over design, they were fed hay of the 3 treatments (native (NAT), soaked (SOA), or steamed (STE)) during an adaptation period of 5 days. On days 6 and 7, horses were equipped with a modified halter (Figure 1) and allowed to consume 2 kg OM of the hay in question (placed directly on the stable ground) for 1 h during the morning meal and the following chewing parameters were detected: chewing rate (CR) in g DM/min, chewing frequency (CFR) in chewing cycles (CC)/s and chewing intensity (CI) in CC/kg DM. This procedure was selected to avoid too much residuals during the test meals (and a mixing with straw) and to calculate the chewing parameters with a defined hay portion. Normally the horses were not able to ingest the amount of 2 kg OM within 1 h time, which is important for unaffected chewing parameters until the end of measuring time. Afterwards, the horses received the residuals of the morning meal. Additionally, the water intake per horse and day (24 h) was measured daily at the same time (8:00 a.m.) with separate water meters per box (L/d).

A sub-sample of the native hay fed to the horses in the in vivo trial was used for the investigations at the laboratory scale. For the soaking treatment, the hay (approximately 4 kg OM) was placed in a plastic tube (approximately 82 L holding capacity, water temperature 12–15 °C) for 15 min and then drained for 10 min. Steaming was also conducted by the use of a common hay steamer (Haygain HG One+, Farm & Stable, West Sussex, UK). The hay (approx. 4 kg OM) was placed into the container and steamed for 60 min to a target temperature of 80–100 °C. The subsequent storage was conducted at 2 different temperatures (10 and 25 °C) and for 3 different time frames (6, 12 and 24 h). Approximately 100 g OM of hay (native and treated) was placed separately on plastic shells (length × width × depth: 55.5 × 37 × 4 cm; maximal 15 cm swath height) and placed in dry cabinets for the above-mentioned conditions. Each variety was arranged in three replicates. The storage temperatures were chosen as an example of colder and warmer environmental conditions. Time frames were deduced from the feeding management in larger barns, in which the hay is normally prepared for the next meal (e.g., soaking in the evening for the morning meal). Subsequently after the individual treatment, the hay sample in question was divided, one part was used for the detection of nutrients and microbial counts, the other stored under the above-mentioned conditions and analyzed afterwards.

The hay was analyzed for dry matter, proximate nutrients (including macro and trace elements), amino acids, neutral detergent insoluble crude protein (NDICP), and microbial counts according to official methods (Association of German Agriculture and Research Institutes (VDLUFA), 2012, 2016 [26]; methods No. 3.1, 4.1.1, 4.11.1, 4.13.1, 5.1.1.B, 6.1.1, 6.5.1, 6.5.2, 8.1, 10.1.1, 10.2.1, 10.3.1, 10.4.1, 10.6.1, 11.1.2, 11.4.2, 11.5.2, 28.1). From proximate nutrients, the content of ME and from NDICP, contents of precaecal digestible (pcd) crude protein and amino acids (AAs) were calculated according to Kienzle and Zeyner (2010) [27] and Zeyner et al. (2015) [28], respectively, as recommended by the German Society of Nutrition Physiology (GfE, 2014) [25]. ME contents were additionally calculated with respect to renal energy losses adjusted to the nitrogen content of the hay [29]. The precaecal digestibility (pcD) of CP was calculated as follows: pcD [%] = (pcdCP × 100)/CP, with CP and pcdCP in grams per kilogram of DM. The WSCs including fructans in the hay were analyzed via the chromatographic method according to Pavis et al. (2001) [30]. 

**Figure 2:**
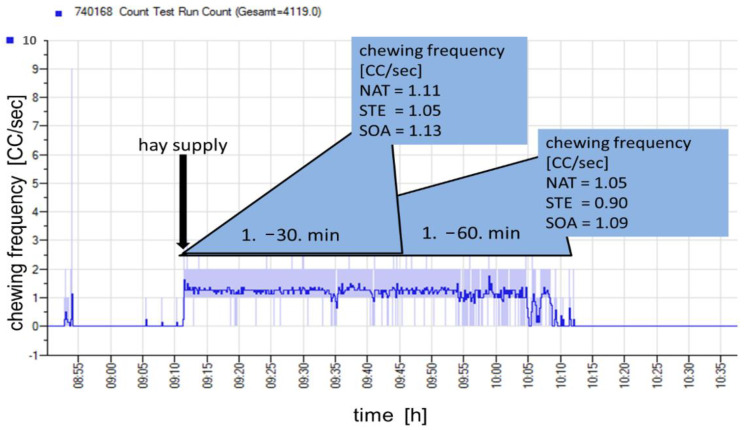
Data sheet from chewing frequencies detected during hay intake. CC, chewing cycle; NAT, native hay; SOA, soaked hay; STE, steamed hay.

FI and chewing parameters were measured using a common halter for horses prepared with a rubber tube below the mandible (Figure 1). For accurate measurement, the halters were validated before the test period, where hand counters were used to adjust sensitivity. This was performed separately for each horse. Pressure differences were detected via a pressure sensor (MPL-503, Conrad Electronics SE, Hirschau, Germany) and saved in a data logger as CF in counts per logging interval (per second). Using the software Tinytag Explorer (version 4.8, Gemini Data Loggers Ltd., Chichester, UK) the data from the logger were transferred in the data sheets (Figure 2) from which the chewing parameters (CR, CFR and CI) were calculated.

The microbial analysis was performed according to the German official methods (VDLUFA, 2012, method No. 28.1, [26]) where the microbial counts were classified into groups and the microorganisms identified in these groups were considered to be representative of typical or spoilage-indicating bacteria, molds and yeast (Table 1). Therefore, 20 g OM of the hay (native and treated) was incubated in 380 mL starting suspension (containing sodium dihydrogen phosphate dihydrate, disodium dihydrogen phosphate dihydrate, sodium chloride, peptone, Tween^®^80, and water) for 20 min under continuous shaking (stomacher). Afterwards, a serial dilution was produced beginning from 1:20 (first dilution) to 1:100 for the next 4 steps (until 10^−6^). Counts of yeasts and molds were detected using rose Bengal agar (with chloramphenicol and tergitol) and dichloran glycerin agar. The plates were incubated at 25 °C for 3 d and subsequently stored at room temperature (RT) for 3 d prior to the enumeration. Mesophilic aerobic bacteria were determined using tryptose agar with triphenyl tetrazolium chloride (TTC) and incubated at 30 °C for 2 d with subsequent storage at RT for 3 d before the colonies were counted.

The statistical evaluation of the FI and chewing parameters as well as the daily water intake was performed according the following mixed model (SAS 9.4, SAS Inc. Cary, NC, USA) with treatment (native, soaked, and steamed) as the fixed effect and animal as the random effect:

y_ij_ = µ + treat_i_ + animal_j_ + e_ij_

where y_ij_ = measurement of the trait of interest (FI and chewing traits as well as daily water intake) for the ith level of treatment and the jth animal where µ is the intercept, treat_i_ is the ith level of treatment for the three levels native (i = 1), soaked (i = 2), and steamed (i = 3), animal_j_ is the random animal effect (j = 1,…,6 for the six horses), and e_ij_ is the random residual effect for the corresponding measurement y_ij_

For statistical analysis, the raw data for microbial counts were transformed logarithmically according to the following formula: y = log_10_(y_raw_ value + 1). For each variety (treatment, storage duration and temperature), 3 samples were used individually for microbial analysis (in each a double determination was conducted according to the official method) and pooled for chemical analysis. Data for proximate nutrients are reported as grams per kilogram on a DM basis. The results for microbial counts (bacteria, yeasts and molds) are expressed as mean colony forming unit per gram (CFU/g).

The differences in microbial counts were analyzed using SAS 9.4 (SAS Inc. Cary, NC, USA) with treatment, storage time and temperature as factors using the mixed procedure that considered one, two, or three factors and all interactions based on ANOVA. One of the following models was used:

M1: y_ir_ = µ + treat_i_ + e_ir_

M2: y_ijr_ = µ + treat_i_ + temp_j_ + (treat × temp)_ij_ + e_ijr_

M3: y_ijkr_ = µ + treat_i_ + temp_j_+ time_k_ + (treat × temp)_ij_ + (treat × time)_ik_ + (temp × time)_jk_

+ (treat × temp × time)_ijk_ + e_ijkr_

where y_ir_ = measurement of the trait of interest (microbial counts for the ith level of treatment, i = 1, 2, 3 and rth repeated sample (r = 1, 2, 3)), M1 (one-way), used for storage duration of 0 h.

y_ijr_ = measurement of the trait of interest (microbial counts for the ith level of treatment, i = 1, 2, 3 and the jth level of temperature, j = 1, 2 (10 °C/25 °C), and the rth repeated sample), M2 (two-way), used for storage duration of 24 h.

y_ijkr_ = measurement of the trait of interest (microbial counts for the ith level of treatment, i = 1, 2 (soaked/steamed), the jth level of temperature, j = 1, 2 (10 °C/25 °C), the kth level of storage duration, k = 1, 2, 3 (6 h/12 h/24 h) and the rth repeated sample), M3 (three-way), used for the given levels for the three fixed effects.

µ = intercept (M1, M2, M3)

where treat_i_ is the ith level of treatment for the three levels native (i = 1), soaked (i = 2), or steamed (i = 3) (M1, M2),

treat_i_ is the ith level of treatment for two levels soaked (i = 1), or steamed (i = 2) (M3),

temp_j_ is the jth level of temperature for the two levels 10 °C (j = 1), and 25 °C (j = 2) (M2, M3),

time_k_ is the kth level of duration storage for the three levels 6 h (k = 1), 12 h (k = 2), and 24 h (k = 3) (M3),

e_ir_ (M1), e_ijr_ (M2), e_ijkr_ (M3) = random residual effect for the corresponding measurement y_ir_ (M1), y_ijr_ (M2), y_ijkr_ (M3).

Differences in means were considered significant at *p* ≤ 0.05. 

## 3. Results

The meadow hay (primarily composed of *Graminae*) was a first cut from middle Eastern Germany (end of blossom).

### 3.1. Proximate Nutrients

The hay had a common crude protein content (89 g/kg DM) and high crude fiber content (356 g/kg DM, Table 2). The concentration of total WSC was 76 g/kg DM (Table 3) with a fructan content of 37.2 g/kg DM, and a total content of mono- and dimeric sugars of 38.9 g/kg DM. The soaked hay had a DM content of 310 g/kg, whereas the steamed hay had a DM content of 380 g/kg. The crude fiber content varied after soaking and steaming in comparison to the native hay (soaked: 418, steamed: 349, and native: 356 g/kg DM; Table 2), and subsequent storage led to only slight variations (Table 2). Equally, the aNDFom content differed after the treatment (native: 618, soaked: 707, and steamed: 702 g/kg DM) and, except for one timeframe (steamed hay, 6 h at 25 °C), there were only slight variations during storage (Table 2). This was also applicable to the ADFom and ADL contents (Table 2). The acid ether extract (AEE) content was nearly similar for the native and steamed hay (Table 2), but slightly different in the soaked hay (Table 2). Soaking and steaming slightly varied the crude ash content in comparison to the native hay (soaked: 55, steamed: 71, and native: 92 g/kg DM; Table 2). The content of pdcCP varied in the native hay (50 g/kg DM) compared to the steamed hay (33 g/kg DM) and the soaked hay (42 g/kg DM). The content of total WSCs differed between the treatments from the native hay (native: 76, soaked: 67, and steamed: 81 g/kg DM; Table 3). The Ca content was slightly higher in the native hay in comparison with the treated hay samples, whereas the P concentration was nearly equal in the native and steamed hay, but less in the soaked variety. K and Na were lower in the soaked hay. Concerning the trace elements (Table 4), the treatment effect on Zn was negligible, and a small increase for Cu and Mn following treatment was observed.

### 3.2. Feed intake Parameters

During the study, neither the bw of the horses (537 ± 41.3 kg, *p* > 0.05) nor the BCS (5.6 ± 0.36/9) changed. All the horses accepted the diets very well, even though some horses seemed to develop a preference. Firstly, the increase in water vapor from the steamed hay led to restrained ingestion behavior at day 1 of the adaptation time, but no longer affected the feed intake behavior from day 2 onwards. The soaked hay seemed to have a reduced palatability, because the horses showed a restrained feed intake. The treatment had no effect on CFR if we only consider the first 30 min of ingestion time. All the treatments induced CFR in the range of ~1.1 CC/s (Table 5). Prolongation of the measuring time up to 1 h shifted the results, as follows: soaked hay induced a tendentially higher CFR (1.09 CC/s) compared to steamed hay (0.90 CC/s, *p* = 0.08), and was in the range of the native hay (1.05 CC/s, Table 5). This observation was more pronounced considering the CI illustrated by the higher chewing cycles per kilogram dry matter for the soaked hay (3537 CC/kg DM) compared to native (2622 CC/kg DM) or steamed hay (2521 CC/kg DM, *p* < 0.05). This was also true for the consumption rate represented by a higher amount of ingested dry matter per minute for native (24.4 g DM/min) and steamed (21.5 g DM/min) hay compared to the soaked hay (19.5 g DM/min), (*p* < 0.05, Table 5). In addition to the FI parameters, the intake of tap water per day was also significantly affected. Here, the ingestion of soaked hay led to a significantly lower water intake (21.6 L/d) compared to those during the feeding of steamed (30.3 L/d) or native hay (32.3 L/d, *p* < 0.05, Table 5). 

### 3.3. Microbial Counts

The native hay contained a high count (compared to the recommended benchmark for native hay, Table 1) of typical bacterial species (29 × 10^6^ CFU/g), which was lower in the treated hay (soaked, 0 × 10^6^ CFU/g; steamed, 20 × 10^6^ CFU/g; *p* > 0.05; Table 6).Typical fungi species were detected in the native hay, which were, however, within the recommended benchmark (64 × 10^3^ CFU/g; Table 1 and Table 6). The soaked hay contained a lesser amount of these typical fungi species (50 × 10^3^ CFU/g; *p* > 0.05), but the content increased during storage (6 h at 25 °C: 87 × 10^3^ CFU/g; *p* > 0.05; Table 6), whereas nearly no typical fungi were detected in the steamed hay (except for 6 h storage at 10 °C: 11 × 10^3^ CFU/g, *p* > 0.05; Table 6). Storage at 10 °C, over a period of 24 h, decreased the content of typical fungi in the hay from both treatments compared to the native one (native: 62; soaked: 36; and steamed: 0 × 10^3^ CFU/g; *p* < 0.05; Table 6). Conversely, following 24 h storage at 25 °C, typical fungi increased exclusively in the soaked hay (native: 24 and soaked: 59 × 10^3^ CFU/g; *p* < 0.05; Table 6). Spoilage-indicating fungi species were detected primarily in the soaked hay, in which group five fungi were only present after storage, whereas group six fungi were already present after treatment (Table 6). After a 24 h storage period, group six fungi were also detected in the native hay. The native hay contained a high amount (compared to the recommended benchmark, Table 1) of yeasts (103 × 10^3^ CFU/g), which were reduced by storage at a lower environmental temperature (24 h at 10 °C; 28 × 10^3^ CFU/g), but nearly not affected by higher temperatures (24 h at 25 °C; 94 × 10^3^ CFU/g; Table 6). The soaked hay also had a high (within the recommended benchmark, Table 1) count of yeasts (90 × 10^3^ CFU/g; Table 6), which increased with storage time (up to 24 h) and with increasing storage temperature (24 h at 10 °C; 515 × 10^3^ CFU/g; *p* > 0.05; 24 h at 25 °C; 545 × 10^3^ CFU/g; *p* < 0.05; Table 6). In the steamed hay, no spoilage fungi or yeast were found (*p* < 0.05), and only one typical mold was detected (Table 6).

## 4. Discussion

The findings of this study indicate that the soaking and steaming of hay have an impact on the feed intake patterns of horses. Furthermore, the treatment and subsequent storage of hay influence its hygienic quality.

The results regarding FI and chewing parameters are in the range of those obtained in previous studies, where CFR ranged from 1.0 to 1.3 CC/s, CR from ~40 to 70 min/kg DM, and CI from ~2.000 to 5.100 CC/kg DM were reported for several types of forages (hay, alfalfa, and meadow hay) [4,5,31,32,33]. Those studies addressed differences between forage that were caused by individual characteristics, rather than those effected by soaking or steaming. In this recent study, treatment significantly affected the CR and CI of horses, which was mainly observed for the soaked hay, which had the lowest DM content. Therefore, the altered DM content, but also the associated changed tactile stimuli during ingestion, might be an important issue in this regard. 

Hay treatment affected not only the hays’ water content, but also the horses’ voluntary intake of water, and thus the total water intake. Calculated for a fictitious leisure horse with a body weight corresponding to the mean body weight of the horses subjected to the current study (536 kg, which is 111 kg bw^0.75^) and a recommended hay intake according to their maintenance energy requirement (0.52 MJ ME/kg bw^0.75^ [25]), this horse would need 58 MJ ME/d from hay, with 5.8 MJ ME/kg DM (native hay in the current study) being 10 kg DM of hay to cover the energy need at the maintenance level. Considering the corresponding DM contents of the native and treated hay (native: 920; steamed: 680; and soaked: 310 g/kg), the horse had a moisture intake of 0.8, 6.9, and 3.2 L/d via the provided native, steamed, and soaked hay, respectively. In addition to the tap water intake of 32.3, 30.3, and 21.6 L/d, accompanying the intake of about 10 kg DM from native, steamed and soaked hay, respectively, the total water intake would amount to 33.1, 33.5, and 28.5 L/d from native, steamed and soaked hay, respectively. This indicates no potential to enhance the total moisture intake by treating hay with water or steam. The high moisture content of the soaked hay is unchanged, unfortunately, in the case of freezing temperatures. 

The aNDFom content also changed after treatment (ranging from ~620 g/kg DM in native to ~700 g/kg DM in steamed or soaked hay), which might be a relative elevation, due to a partial wash out of soluble nutrients. Depending on the storage duration, the aNDF content decreased to values close to the starting level. The proportion of structural fiber (NDF) in the DM content of forage can vary between 33% (e.g., young grasses) and ~80% in straw, which increases saliva production, according to Ellis et al. (2005) [34]. More intensive chewing can mitigate or at least decrease tooth problems because of the more abrasive characteristics of forage compared to concentrates and their positive effect on the tooth surfaces [2]. Equines need these feedstuffs, because their teeth are adapted to a highly abrasive feed [35]. In this study, the horses chewed the soaked hay with a significantly elevated frequency (increase by nearly 1000 CC/kg DM for steamed vs. native hay) and additionally ingested it at a significantly lower CR. This observation would normally be viewed as an advantage, because we want horses to spend more time on FI and chew at high intensities during the ingestion of their daily rations. Especially in the present study, this more intensive chewing combined with a prolonged CR was only observed for the soaked hay, regardless of the nearly equal NDF contents (Table 2). Therefore, it seems possible that this was caused by reduced palatability and lesser nutritional value, even though the slightly reduced content of WSCs may have additionally reduced the acceptance. However, it should be considered that soaking and steaming alter these physical and haptic properties of the hay, with possible consequences on the chewing behavior. So, wet material is not as brittle, and thus cannot be crushed as easily by the chewing process. More chewing cycles might be necessary to process bite. Steaming, in this regard, might partly remove hemicelluloses, as well as melt and agglomerate depolymerized lignin, which also alters the “structure”. This was demonstrated with wood chips at test temperatures of 200 °C and above [36]. In this study, the patterns of chewing steamed hay were close to those measured for native hay.

To meet the energy requirements of horses, the recommendations from the GfE (2014 [25]; 0.52 MJ ME/kg bw^0.75^) were used to calculate the daily rations in the current study. For this, a horse with a bw of 536 kg would receive 10 kg DM of the used native hay per day, which would additionally meet the daily requirements of pcdCP (315 g) and pcd amino acids (AAs), such as lysine (13 g), methionine, and cystine (9 g), as well as threonine (16 g; Table 2). A limiting factor of the present study was that only one hay sample per treatment was used for the analysis of the proximate nutrients, which subsequently limited the estimation regarding possible treatment effects; soaking the hay led to a mean loss of ~20% (15–23%) of ME, pcdCP, and pcdAA. The pcD of CP and AA (56% in native hay) was clearly reduced after steaming (47%), but not after soaking (58%). Bochnia et al. (2021) [23] detected similar results concerning the reduction in CP and pcd CP after soaking hay after 15 min. In this study, however, 120% and 140% more soaked and steamed hay, respectively, would be required to cover the needs for ME, pcdCP, and pcdAA. Therefore, it is recommended to increase the daily amount of treated hay provided, to compensate for these losses or decreases in pcD, respectively. The problem posed for feeding practice is that the actual treatment-induced loss cannot be predicted, and so the decline in pcdCP and pcdAA may be higher or lower than that of ME. However, a particular interesting result is the substantial drop in the pcD of CP and pcD of the investigated AAs following steaming. Heating and the availability of water as well as nitrogen and simple sugars from the feed during the steaming process may affect the protein structure, as well as the availability of AAs. Special heat treatments may stabilize feed protein structures [37,38,39,40,41] with complex denaturation reactions, and the formation of Maillard polymers [42,43,44,45,46] in fibrous feeds, such as grasses [42]. An actual decrease in pcD CP and pcD AA following steaming, reported here from a single sample only, would be an important issue for horse feeding, so we recommend improving this with a larger number of samples and parallels.

Steamed hay can achieve energy contents similar to those of soaked hay, but the pcdCP is much lower. A decrease of >30% for pcdCP (native: 50; steamed: 33; Table 2) and pcdAA (e.g., lysine, native: 1.77; steamed: 1.33) after steaming led to the recommendation to increase the offered hay portion by at least this amount, to meet the requirements for pcdCP and pcdAA. In detail, this means that horse owners must steam a portion of ~12 kg OM hay (=140%) for a 500 kg bw horse, to prevent weight loss during the usage of steamed hay. In the present study, we observed no effect on bw, due to the short time for adaptation and test days, because the whole study only lasted 3 weeks. In practice, long-term effects can induce weight loss, but also deficiency in other essential nutrients (macro and trace elements) if the horses received portions of soaked or steamed hay that do not take the wash-out effect into account.

The DM reduction was the highest in the soaked hay in comparison to the steamed hay, which coincides with previous findings [9,21]. Concerning the storability, a higher moisture content of the hay favors the formation of mold and spoilage-indicating bacteria [9,19]. Viable microorganisms were only detected in the subsequent stored soaked hay, but not in the steamed hay, indicating a serious impact of the treatment method (and treatment temperature). The target DM content for stable preserved hay is above 850 g/kg, whereas a lower content increases the risk of microbial growth, as well as spoilage [9,19,47]. Both treatments led to a higher moisture content, which is why the hay was no longer stable in storage. If not fed immediately after treatment, the high moisture content might result in the proliferation of microorganisms, particularly spoilage-indicating microbes, especially at high environmental temperatures. This, however, may only apply to soaked hay, because streaming led to a substantial reduction in the counts of viable microbes.

The native hay contained a slightly higher (in relation to the recommended benchmark for hay) content of typical bacteria and yeasts. Furthermore, the content of typical fungi was within the recommended range and no spoilage-indicating fungi or molds were detected. Soaking decreased the content of typical bacteria (by 100%), fungi (by 22%), and yeasts (by 11%), which mostly coincides with findings in the literature [16,19]. Moreover, after soaking, spoilage fungi were detected. The concentration of yeasts increased with storage time and a higher ambient temperature. In contrast, steaming reduced the content of typical bacteria by 32% and by 100% for the typical/spoilage-indicating mold, fungi, and yeast. This coincides with previous findings in the literature, where the reduction in bacteria was described as being even higher (reduction of 98–99%; [14,17,48]). Nonetheless, the bacteria as well as fungal spores are not physically removed from the hay, and are still present (dead or alive) [9,16]. Furthermore, it is unknown if spores were washed out or remained in the hay after the treatment (soaking or steaming) [12]. Perhaps the microbial spores are physically washed out during soaking, but not during steaming treatment. The effect of these spores (maybe with germination ability), in terms of allergenic potential, the forage taste, or the development of gastrointestinal disorders, is unknown. Some authors identified negative consequences due to the ingestion of high amounts of endotoxins derived from bacteria [12,16] or mycotoxins derived from fungi that are capable of causing toxicity [47]. Nonetheless, steaming of the hay is recommended if the native hay contains high amounts of bacteria or fungi/yeast [48]. The subsequent storage of the steamed hay led to no further proliferation of bacteria, fungi, yeast, or mold, except for the measurement after 6 h. Conversely, the soaked hay contained, to a higher degree with increasing storage time and temperature, a higher content of typical bacteria (group one), spoilage mold/fungi (groups five and six), and yeasts (group seven). High temperatures in association with a longer storage duration led to increasing amounts of typical/spoilage-indicating yeasts. Overall, the risk of metabolic disorders, more precisely colic, increases with decreasing hygienic quality (associated with, e.g., a high amount of yeasts) [49].

Considering the reduction in airborne and alveolar particles, this study confirms former results that both treatments seem to be effective [12,13,16,17,18]. However, steaming might be preferred, due to the reduction or even avoidance of the waste water that poses environmental issues [18,50]. In the handling of horses suffering from equine asthma, it is further recommended not to let the soaked hay dry out, because the airborne particles might enter the hay [13]. Furthermore, our findings confirm that the storage of the soaked hay results in the proliferation of spoilage-indicating microorganisms, which also poses risks in terms of equine health.

## 5. Conclusions

In general, high-quality native hay is the recommended feed for horses and does not need to be treated. If only hygienically poor hay is available, or there is a relevant health predisposition in the horse (e.g., equine asthma), the soaking or steaming of hay might be helpful. This, however, needs to be considered in the context of any subsequent storage. Soaking hay reduces microbial cell counts, but a long storage duration in combination with warm temperatures amplifies the proliferation of spoilage-indicating molds and yeasts in the soaked hay. Steaming is very effective concerning the improvement in hygienic quality, and steamed hay is less susceptible to spoilage during storage. Soaking is thus only recommended if the treated hay is fed immediately. Soaking led to a higher reduction in water-soluble nutrients than steaming, which can be positive or negative, depending on the kind of nutrient and the health precondition of the horse in question. Dietary compensation (energy, amino acids, and minerals) might be required when soaked and, to a lesser extent, when steamed hay are fed, but this is difficult to implement in practice. The longer eating time and the higher chewing intensity for soaked hay compared to native or steamed hay had a positive impact on the dental and gastrointestinal health, but this might be biased by a reduced palatability of the latter.

## Figures and Tables

**Table 1:** Classification of the microbial species to indicator groups *.

Group	Indicator Species	Classification	Benchmark Native Hay (CFU/g)
1	yellow bacteria germs	typical microbial species	30 × 10^6^
	*Pseudomonas* spp. *Enterobacteriaceae*		
	other typical bacteria		
2	*Bacillus* spp. *Staphylococcus* spp.	spoilage-indicating microbial species	2 × 10^6^
	*Micrococcus* spp.		
3	Streptomycetes	spoilage-indicating microbial species	0.15 × 10^6^
4	sooty mold fungi *Verticillum* spp.	typical microbial species	200 × 10^3^
	*Acremonium* spp. *Fusarium* spp. *Aureobasidium* spp. other typical fungi		
5	*Aspergillus* spp. *Penicillum* spp. *Scopulariopsis* spp. *Wallemia* spp.	spoilage-indicating microbial species	100 × 10^3^
	other spoilage fungi		
6	*Mucorales*	spoilage-indicating microbial species	5 × 10^3^
7	all yeast species	typical or spoilage species	150 × 10^3^

* According to the official method of the VDLUFA (2012) [26].

**Table 2:** Proximate nutrients of the native and treated hay.

Treatment	Storage Condition		DM	CA	CP	pcdCP	pcd Lys/Met + Cys/Thre	AEE	CF	aNDFom	ADFom	ADL	ME	MEmod ^1^
	Time	°C	g/kg	g/kg DM									MJ/kg DM	
native		10	920	92	89	50	1.77/1.06/1.67	11	356	618	361	62	5.8	5.8
soaked	0 h		310	55	73	42	1.79/0.96/1.75	7	418	707	471	72	5.2	5.2
	6 h	10	320	70	99			10	385	679	444	73		
	12 h	10	330	69	100			10	421	683	497	80		
	24 h	10	450	65	86			7	420	678	447	76		
	6 h	25	350	65	85			9	389	683	439	69		
	12 h	25	360	61	88			12	469	704	506	86		
	24 h	25	610	103	96			9	408	660	442	81		
steamed	0 h		680	71	70	33	1.31/0.73/1.32	6	394	702	465	64	5.4	5.4
	6 h	10	840	73	83			6	386	692	467	75		
	12 h	10	840	95	80			9	386	666	460	71		
	24 h	10	920	81	86			6	389	672	448	77		
	6 h	25	830	122	106			7	395	581	393	70		
	12 h	25	680	76	96			10	389	668	436	70		
	24 h	25	940	91	101			7	400	653	429	80		

ADFom, acid detergent fiber; AEE, acid ether extract; aNDFom, neutral detergent fiber; DM, dry matter; CA, crude ash; CP, crude protein; CF, crude fiber; pcdCP, precaecal digestible crude protein. ^1^ According to Kuchler et al. (2020) [29].

**Table 3:** Measured contents of water-soluble carbohydrates in the native and treated hay.

Treatment	Storage Condition		Fructan	Saccharose	Glucose	Fructose	WSC
	Time	Temperature	g/kg DM				
native	(h)	(°C)	37	6	11	21	76
soaked	0		21	8	16	23	67
	6	10	15	3	7	18	42
	12	10	11	4	10	14	39
	24	10	14	2	6	15	37
	6	25	22	3	9	21	54
	12	25	15	4	4	11	35
	24	25	16	6	4	8	34
steamed	0		31	18	13	19	81
	6	10	21	19	9	14	64
	12	10	17	15	11	16	59
	24	10	21	20	10	15	67
	6	25	19	12	6	11	49
	12	25	17	13	10	15	54
	24	25	17	16	7	12	52

DM, dry matter; WSCs, water-soluble carbohydrates (sum of mono- and dimeric sugars and fructans).

**Table 4:** Measured contents of macro and trace elements in the native and treated hay.

	*p*	Ca	K	Na	Mg	Zn	Mn	Cu	Fe
Variety	g/kg DM					mg/kg DM			
native	2.1	5.7	18.7	0.2	0.7	16.6	19.2	5.3	139.1
soaked	1.8	5.2	10.3	0.9	1.0	15.9	32.0	7.3	157.4
steamed	2.0	5.2	17.6	1.1	1.1	16.4	31.5	9.2	88.0

DM, dry matter.

**Table 5:** LSmeans (± SE) of chewing parameters and water intake (L/d) in horses after feeding native, soaked and steamed hay.

Treatment			
Item	Native	Soaked	Steamed
chewing frequency (CC/s)	1.05 ±0.078	1.09 ^#^ ±0.078	0.90 ^#^ ±0.078
chewing frequency ^30^ (CC/s)	1.11 ±0.053	1.13 ±0.053	1.05 ±0.053
chewing intensity (CC/kg DM)	2622 ^b^ ±217.3	3537 ^a^ ±217.3	2521 ^b^ ±217.3
consumption rate (g DM/min)	24.4 ^a^ ±2.89	19.5 ^b^ ±4.94	21.5 ^a^ ±2.04
water intake (L/d)	32.3 ^A^ ±2.13	21.6 ^B^ ±2.13	30.3 ^A^ ±2.13

CC, chewing cycles; chewing frequency ^30^, first 30 min during intake; DM, dry matter. ^a,b^ Means in the same row per group are statistically significant different from each other (*p* < 0.05). ^A,B^ Means in the same row per group are statistically significant different from each other (*p* < 0.01). ^#^ *p* = 0.08.

**Table 6:** Detected microbial counts (in CFU/g; Lsmeans ± SE) in the native and treated hay.

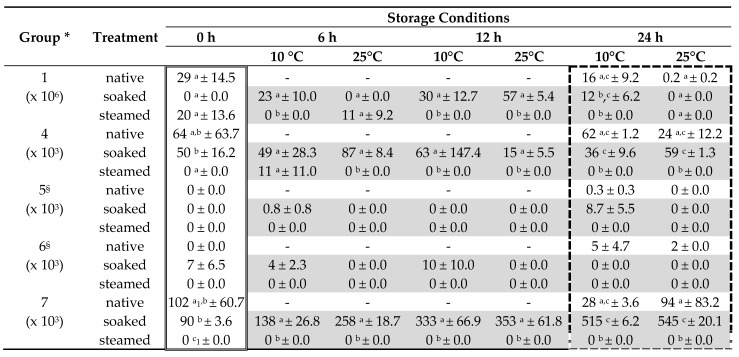

* Group according to Table 1. - Not examined. ^§^ Data were not statistically analyzed because of the sparse amount of values (detected microbial counts) available. ^a,b,c^ Means in the same column per group are statistically significant different from each other (*p* < 0.05); logarithmic values were compared statistically; the following statistical model was used: double-lined frame = M1 (one-way), dashed-lined frame = M2 (two-way), highlighted in grey = M3 (three-way). ^a^_1_^,c^_1_ *p* = 0.0501.

## Data Availability

All data are included in this article.
